# Propyl gallate induces cell death in human pulmonary fibroblast through increasing reactive oxygen species levels and depleting glutathione

**DOI:** 10.1038/s41598-024-52849-z

**Published:** 2024-03-05

**Authors:** Woo Hyun Park

**Affiliations:** Department of Physiology, Medical School, Jeonbuk National University, 20 Geonji-Ro, Deokjin, Jeonju, Jeollabuk 54907 Republic of Korea

**Keywords:** Cell biology, Molecular biology

## Abstract

Propyl gallate (PG) exhibits an anti-growth effect on various cell types. The present study investigated the impact of PG on the levels of reactive oxygen species (ROS) and glutathione (GSH) in primary human pulmonary fibroblast (HPF) cells. Moreover, the effects of N-acetyl cysteine (NAC, an antioxidant), l-buthionine sulfoximine (BSO, a GSH synthesis inhibitor), and small interfering RNA (siRNAs) against various antioxidant genes on ROS and GSH levels and cell death were examined in PG-treated HPF cells. PG (100–800 μM) increased the levels of total ROS and O_2_^**·**−^ at early time points of 30–180 min and 24 h, whereas PG (800–1600 μM) increased GSH-depleted cell number at 24 h and reduced GSH levels at 30–180 min. PG downregulated the activity of superoxide dismutase (SOD) and upregulated the activity of catalase in HPF cells. Treatment with 800 μM PG increased the number of apoptotic cells and cells that lost mitochondrial membrane potential (MMP; ΔΨ_m_). NAC treatment attenuated HPF cell death and MMP (ΔΨ_m_) loss induced by PG, accompanied by a decrease in GSH depletion, whereas BSO exacerbated the cell death and MMP (ΔΨ_m_) loss without altering ROS and GSH depletion levels. Furthermore, siRNA against SOD1, SOD2, or catalase attenuated cell death in PG-treated HPF cells, whereas siRNA against GSH peroxidase enhanced cell death. In conclusion, PG induced cell death in HPF cells by increasing ROS levels and depleting GSH. NAC was found to decrease HPF cell death induced by PG, while BSO enhanced cell death. The findings shed light on how manipulating the antioxidant system influence the cytotoxic effects of PG in HPF cells.

## Introduction

Propyl gallate (3,4,5-trihydroxybenzoic acid propyl ester; PG) has been widely used for conserving food and cosmetics and stabilizing pharmaceutical preparations^1^. PG has also been shown to have many health benefits as an antioxidant^2,3^, a chemopreventive agent^4^, and an anti-inflammatory agent^5^. For instance, PG upregulates the activity of superoxide dismutase (SOD) and catalase (CAT) in HeLa cervical cancer cells^6^. PG effectively defends hepatocytes against lipid peroxidation^2^. PG has similar potency as vitamin E and is even more effective than some water-soluble antioxidants such as ascorbic acid^2^. At low concentrations (nM to μM), PG functions as a SOD mimic, providing protection to cultured lens epithelial cells against hydrogen peroxide (H_2_O_2_) insult^7^. Several studies have proposed the mechanism underlying the action of PG: PG binds to transition metals, eliminates the superoxide anion (O_2_^**·**−^) or the hydroxyl radical (^**·**^OH), and activates intracellular signaling and downstream transcription of specific genes^7–9^. However, other studies have reported that 500 μM PG shows pro-oxidant properties via increasing the amount of 8-oxo-7,8-dihydro-2′-deoxyguanosine (8-oxodG), a characteristic oxidative DNA lesion, in human leukemia cell line HL-60^10^. Treatment with PG (500–2000 μM) induces cytotoxicity in freshly isolated rat hepatocytes, leading to mitochondrial damage and a reduction in intracellular ATP, adenine nucleotide pools, glutathione (GSH), and protein thiols ^11^. PG also inhibits respiration and nucleic acid synthesis in microorganisms^12^. Antioxidative and cytoprotective properties of PG can be transformed to pro-oxidative, cytotoxic, and genotoxic properties in the presence of Cu(II)^13^. Moreover, PG promoted growth of human diploid fibroblasts at a concentration of 10^−8^ M but reduced their growth at a concentration of 10^−6^ M or greater^14^. Hence, to understand the discrepancy among these diverse effects of PG described in the literature, further studies are necessary to examine its function, characteristics, and safety.

Reactive oxygen species (ROS) are primarily H_2_O_2_, ^**·**^OH, and O_2_^**·**−^, which are usually detrimental to cells and tissues. Nevertheless, ROS control many indispensable cellular processes such as cell proliferation, differentiation, and death^15^. Any alteration of the redox status in tissues and cells affects the production or metabolism of ROS. ROS are mainly generated as by-products of mitochondrial respiration, e.g., O_2_^**·**−^, and are produced by specific oxidases such as nicotinamide adenine dinucleotide phosphate (NADPH) oxidase and xanthine oxidase^16^. The major metabolic pathways of ROS require SODs [cytoplasmic (SOD1), mitochondrial (SOD2), or extracellular (SOD3) isoforms], which change O_2_^**·**−^ into H_2_O_2_^17^. Supplementary metabolism by CAT or GSH peroxidase (GPX) produces O_2_ and H_2_O^18^. Notably, the thioredoxin (TXN) system consisting of TXN, TXN reductase (TXNR), and NADPH is crucial for maintaining cellular redox homeostasis^19^. TXN, a thiol reductase, is a potent antioxidant to scavenge ROS^19^. In addition, GSH is a crucial nonprotein antioxidant that regulates cell proliferation, cell survival, and apoptosis^20^, and it is known to protect cells from toxic agents by detoxifying them^21^. Excessive ROS production leads to the so-called oxidative stress, which could progress to cell death by damaging cellular DNA, proteins, and lipids. Oxidative stress has been linked to the pathophysiology of many diseases, especially cancer in many ways^22,23^. Because the generation of different ROS arises during multiple cellular processes and can be either beneficial or detrimental to cells and tissues, normally the redox status is tightly controlled to avoid cellular and tissue injury.

Lung cancer is the leading cause of cancer-related mortality worldwide^24^. A number of novel management approaches are still under development, and the available conventional drugs are limited^25^. Investigation of the molecular mechanisms underlying the cytotoxic effects of therapeutic drugs will thus shed light on the optimal treatment of lung cancer. PG is a synthetic agent that has anti-growth effects in many cell types, including endothelial cells^26,27^, leukemia cells^28^, breast cancer^29^, hepatocellular carcinoma cells^30^, and cervical cancer cells^6,31^. Recently, our group has found that PG treatment, with an IC50 of 800 µM at 24 h, inhibits the growth of lung cancer cells, particularly A549 epithelial adenocarcinoma cells via apoptosis or GSH depletion^32,33^. Fibroblast cells are the most abundant type of cells in the pulmonary interstitium. Pulmonary fibroblast (PF) cells play a central role in restoring and remodeling tissues following lung injuries^34^. Insufficient or excessive proliferation of PF cells can cause inflammation and aberrant tissue function^34^. However, little is known about the cytotoxicological properties of PG on normal primary PF cells.

The present study aimed to investigate the impact of PG exposure (100–1600 μM) on ROS and GSH levels in primary human PF (HPF) cells and to examine the effects of NAC (an antioxidant), BSO (a GSH synthesis inhibitor), and siRNAs against various antioxidant genes on ROS and GSH levels and cell death in PG-treated HPF cells.

## Materials and methods

### Cell culture

The primary HPF cells were purchased from PromoCell GmbH (C-12360, Heidelberg, Germany). As per the catalog information provided by PromoCell GmbH, these primary HPF cells are isolated from human lung tissue, and the passage number is two after thawing. The cells were cultured in RPMI-1640 medium supplemented with 10% fetal bovine serum (FBS; Sigma-Aldrich Co., St. Louis, MO, USA) and 1% penicillin–streptomycin (GIBCO BRL, Grand Island, NY, USA). Cultures were maintained in a humidified incubator containing 5% CO_2_ at 37 °C. For experiments, HPF cells within the passage range of four to eight were utilized.

### Reagents

PG, obtained from Sigma-Aldrich Co. (CAS Number: 121-79-9), is a compound with the molecular formula C10H12O5 and a molecular weight of 212.20. Its purity is specified as greater than or equal to 98% according to HPLC analysis. NAC (CAS Number: 616-91-1) and BSO (CAS Number: 83730-53-4) were also acquired from Sigma-Aldrich Co. NAC was dissolved in 20 mM 4-(2-hydroxyethyl)-1-piperazineethanesulfonic acid (HEPES; pH 7.0) buffer to 200 mM as a stock solution, and BSO was dissolved in distilled water to 100 mM as a stock solution. On the basis of the previous studies^35,36^, cells were pre-incubated with 2 mM NAC or 10 μM BSO for 1 h, followed by treatment with PG at 37 °C for 24 h before assays were performed. Ethanol (0.2%) was used as a control vehicle. All stock solutions were wrapped in foil and kept at − 20 °C.

### Determination of intracellular ROS levels

Intracellular ROS levels were measured using various oxidation-sensitive fluorescent probe dyes purchased from Invitrogen Molecular Probes (Eugene, OR, USA). 2′,7′-dichlorodihydrofluorescein diacetate (H_2_DCFDA; Ex/Em = 495 nm/529 nm) was used for the detection of the total ROS level, including H_2_O_2_, ^**·**^OH, and ONOO^**·**^, as previously described^6,37^. Dihydroethidium (DHE; Ex/Em = 518 nm/605 nm) is highly selective for O_2_^**·**−^ among ROS^6,37^. Mitochondrial O_2_^**·**−^ levels were detected using MitoSOX™ Red agent (Ex/Em = 510 nm/580 nm)^37^. In brief, 1 × 10^6^ cells in 60-mm culture dishes (BD Falcon, Franklin Lakes, NJ) were pretreated with 2 mM NAC or 10 μM BSO for 1 h and then treated with PG at an indicated concentration (100–1600 μM) for 24 h or other indicated time points. Cells were then washed in phosphate buffer saline (PBS) and incubated with 20 µM H_2_DCFDA, 20 µM DHE, or 5 µM MitoSOX™ Red agent at 37 °C for 30 min. Mean DCF, DHE, and MitoSOX Red fluorescence signals were measured using a FAC Star flow cytometer (BD Sciences, Franklin Lakes, NJ). Each ROS level following treatment was expressed as a percentage of those of untreated control cells.

### Detection of the intracellular GSH

Cellular GSH levels were analyzed using 5-chloromethylfluorescein diacetate (CMFDA; Ex/Em = 522 nm/595 nm; Invitrogen Molecular Probes) as previously described^6,37^. In brief, 1 × 10^6^ cells in 60-mm culture dishes (BD Falcon) were pretreated with 2 mM NAC or 10 μM BSO for 1 h and then treated with PG at an indicated concentration (100–1600 μM) for 24 h or other indicated time points. Cells were then washed with PBS and incubated with 5 µM CMFDA at 37 °C for 30 min. CMF fluorescence intensity was determined using a FAC Star flow cytometer (Becton Dickinson). CMF-negative (−) staining cells indicate depletion of GSH in cells. The mean CMF levels in cells, except for CMF-negative (GSH-depleted) cells, were expressed as a percentage of those of control cells.

### Measurement of the activity of the total cellular SOD

The activity of the cellular SOD enzymes was measured using a SOD assay kit-WST (Fluka Co., Milwaukee). WST-1 (2-(4-lodophenyl)-3-(4-nitrophenyl)-5-(2,4-disulfophenyl)-2H-tetrazolium, monosodium salt) yields a water-soluble formazan dye upon reduction with O_2_^**·**−^^6,27^. In brief, 1 × 10^6^ cells were incubated with 800 μM PG for 24 h. Supernatants that contain 30 μg protein were used for measuring SOD activity. Formazan crystal formation was measured at 450 nm using a microplate reader (Synergy™ 2, BioTek Instruments Inc., Winooski, VT, USA).

### Measurement of cellular catalase activity

The activity of cellular catalase enzyme was measured using a catalase assay kit from Sigma-Aldrich Co.^6,27^. In brief, 1 × 10^6^ cells were incubated with 800 μM PG for 24 h. Supernatants containing 30 μg protein were used for measuring catalase activity. The production of a red quinoneimine dye was measured at 520 nm using a microplate reader (Synergy™ 2, BioTek Instruments Inc.).

### Annexin V-FITC/PI staining for detecting cell death

Apoptosis was identified via annexin V-fluorescein isothiocyanate staining (FITC, Life Technologies, Carlsbad, CA; Ex/Em = 488 nm/519 nm) and propidium iodine staining **(**PI, Sigma-Aldrich Co.; Ex/Em = 493 nm/636 nm) as previously described^38^. Briefly, 1 × 10^6^ cells in 60-mm culture dishes (BD Falcon) were pretreated with 2 mM NAC or 10 μM BSO for 1 h and then treated with PG for 24 h. Cells were washed twice with cold PBS and then resuspended in 200 μL binding buffer (10 mM HEPES/NaOH, pH 7.4, 140 mM NaCl, and 2.5 mM CaCl_2_) at a concentration of 5 × 10^5^ cells/mL at 37 °C for 30 min. Annexin V-FITC (2 μL) and PI (1 μg/mL) were added to the solution, and cells were analyzed using a FAC Star flow cytometer (BD Sciences).

### Measurement of MMP (ΔΨ_m_)

The MMP (ΔΨ_m_) was monitored using rhodamine 123 (Sigma-Aldrich Co.; Ex/Em = 485/535 nm), which is a fluorescent, cell-permeable, and cationic dye that favorably enters the mitochondria with high negative MMP (∆Ψ_m_). Depolarization of MMP (∆Ψ_m_) results in the loss of rhodamine 123 from the mitochondria and reduction of the intracellular fluorescence intensity of this dye, as previously described^38^. Briefly, 1 × 10^6^ cells in 60-mm culture dishes (BD Falcon) were pretreated with 2 mM NAC or 10 μM BSO for 1 h and then treated with PG for 24 h. Cells were washed twice with PBS and incubated with rhodamine 123 (0.1 mg/mL) at a concentration of 5 × 10^5^ cells/mL at 37 °C for 30 min. The intensity of rhodamine 123 staining was determined using a FAC Star flow cytometer. Rhodamine 123-negative (−) cells represent HPF cells with MMP (∆Ψ_m_) loss. MMP (ΔΨ_m_) levels in cells without MMP (ΔΨ_m_) loss were expressed as a percentage of levels in control cells.

### Transfection of cells with antioxidant-related siRNAs

siRNA silencing of SOD1, SOD2, CAT, GPX, and TXN was carried out as previously described^39,40^. The non-specific control siRNA duplex [5′-CCUACGCCACCAAUUUCGU(dTdT)-3′], SOD1 siRNA duplex [5′-GAAAACACGGUGGGCCAAA(dTdT)-3′], SOD2 siRNA duplex [5′-CUGGGAGAAUGUAACUGAA(dTdT)-3′], CAT siRNA duplex [5′-CACUGAUUUCACAACAGAU(dTdT)-3′], GPX siRNA duplex [5′-CAAGCUCAUCACCUGGUCU(dTdT)-3′] and TXN siRNA duplex [5′-GCAUGCCAACAUUCCAGUU(dTdT)-3′] were obtained from Bioneer Corporation (Daejeon, South Korea). In brief, 2.5 × 10^5^ cells in six-well plates (Nunc, Roskilde, Denmark) were incubated in RPMI-1640 supplemented with 10% FBS. The next day, cells (at approximately 30–40% confluency) in each well were transfected with the negative control or gene-specific siRNA duplex [80 pmol in Opti-MEM (GIBCO BRL)] using LipofectAMINE 2000 according to the manufacturer’s instructions (Invitrogen, Carlsbad, CA, USA). After 24 h, cells were treated with either PG (800 μM) or solvent control for an additional 24 h. Transfected cells were collected and used for staining with annexin V-FITC/PI and for measuring ROS and GSH levels.

### Statistical analysis

The results represent the mean of two or three independent experiments (mean ± SD). The data were analyzed using Instat software (GraphPad Prism 5.0, San Diego, CA). A Student’s *t*-test or one-way analysis of variance with post-hoc Tukey’s multiple comparison test was used to assess statistical significance, which was defined as *P* < 0.05.

## Results

### Effects of PG on intracellular ROS levels in HPF cells

**Figure 1:**
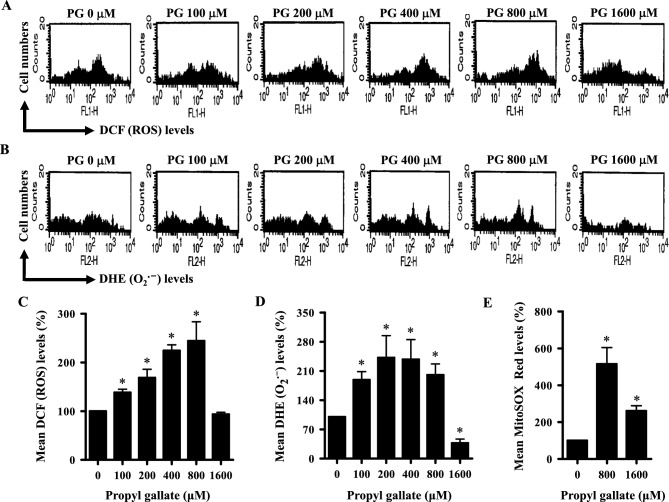
Effects of PG on intracellular ROS levels in HPF cells at 24 h. Exponentially growing cells were treated with the indicated amount of PG for 24 h. Intracellular DCF (ROS), DHE (O_2_^**·**−^), and MitoSOX Red (mitochondrial O_2_^**·**−^) levels in HPF were measured using a FAC Star flow cytometer. (**A**,**B**) Each histogram shows the DCF (ROS) (**A**) and DHE (O_2_^**·**−^) levels (**B**) in PG-treated HPF cells. (**C**,**D**) Graphs indicate DCF (ROS) levels (%) from A (**C**) and DHE (O_2_^**·**−^) levels (%) from B (**D**). (**E**) Graph shows MitoSOX Red (mitochondrial O_2_^**·**−^) levels (%) in HPF cells. *P < 0.05 compared with the untreated control group.

To determine the intracellular levels of ROS in PG-treated HPF cells, H_2_DCFDA dye, DHE dye, and MitoSOX Red dye were used for measuring the levels of total ROS, O_2_^**·**−^, and mitochondrial O_2_^**·**−^, respectively. As shown in Fig. 1A,C, the total intracellular ROS levels were significantly increased in HPF cells treated with 100–800 μM PG at 24 h. Treatment with 800 μM PG increased the total ROS level by about 2.5-fold compared with the baseline level. However, 1600 μM PG did not alter the ROS level in HPF cells (Fig. 1A,C). Intracellular O_2_^**·**−^ levels were also increased in HPF cells treated with 100–800 μM PG at 24 h, and the O_2_^**·**−^ level was significantly decreased in cells treated with 1600 μM PG (Fig. 1B,D). Mitochondrial O_2_^**·**−^ levels were increased in HPF cells treated with 800 and 1600 μM PG at 24 h (Fig. 1E). Specifically, 800 μM PG increased the mitochondrial O_2_^**·**−^ level by about fivefold (Fig. 1E).

**Figure 2:**
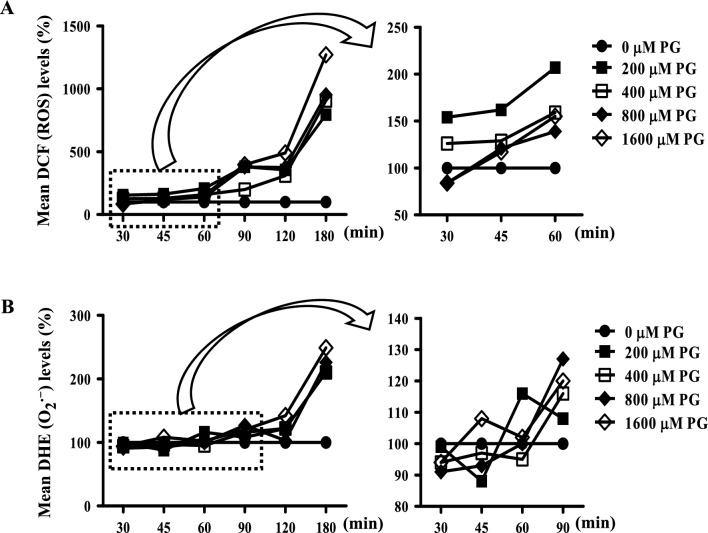
Effects of PG on intracellular ROS levels in HPF cells at the early time points. Exponentially growing cells were treated with the indicated amount of PG for the indicated time points. Intracellular DCF (ROS) and DHE (O_2_^**·**−^) levels in HPF were measured using a FAC Star flow cytometer. (**A**) Graphs show DCF (ROS) levels (%) in HPF cells. The graph on the right shows an enlarged graph with PG incubation at 30–60 min. (**B**) Graphs show DHE (O_2_^**·**−^) levels (%) in HPF cells. The graph on the right shows an enlarged graph with PG incubation at 30–90 min.

At the early time points of PG incubation (30–180 min), all doses of PG (100–1600 μM) tested in this study increased the total ROS levels, with the most dramatic increases appearing at 90 min (Fig. 2A). At 180 min, 800 and 1600 μM PG treatment increased the ROS levels by 10- and 12-fold compared with the baseline levels, respectively (Fig. 2A). By contrast, all doses of PG (100–1600 μM) decreased intracellular O_2_^**·**−^ levels at 30 and 45 min, except that the treatment with 1600 μM PG increased the O_2_^**·**−^ level at 45 min (Fig. 2B). All doses of PG increased O_2_^**·**−^ levels at 90 min and later time points, with the most dramatic increase at 180 min (Fig. 2B).

### Effects of PG on intracellular GSH levels in HPF cells

**Figure 3:**
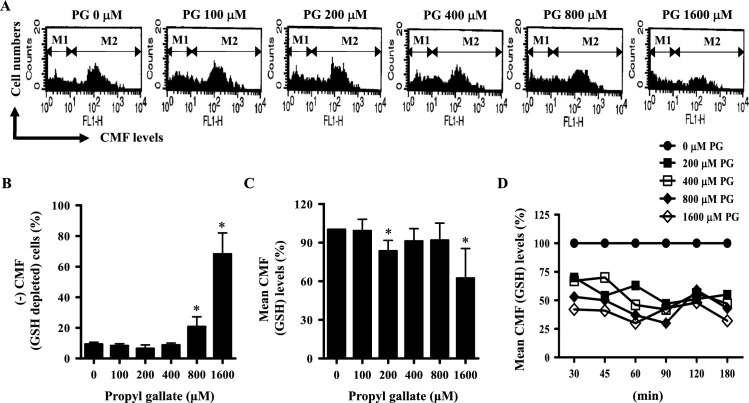
Effects of PG on intracellular GSH levels in HPF cells. Exponentially growing cells were treated with the indicated amount of PG for 24 h or other indicated time points. Intracellular GSH levels were determined using a FAC Star flow cytometer. (**A**) Each histogram shows the CMF (GSH) levels in PG-treated HPF cells at 24 h. M1 indicates CMF-negative (GSH-depleted) HPF cells. M2 indicates cells without GSH depletion. (**B**) Graph shows the percentage of CMF-negative (GSH-depleted) cells derived from M1 regions in (**A**). (**C**) Graph shows mean CMF (GSH) levels (%) derived from M2 regions in (**A**), compared with control cells. (**C**) Graph shows mean CMF (GSH) levels (%) at early time points of 30–180 min, compared with control cells. *P < 0.05 compared with the untreated control group.

Intracellular GSH levels were assessed with CMFDA, which produces a fluorescent signal upon reacting with the reduced form of GSH. When the number of GSH-depleted cells was measured among HPF cells treated with 100–1600 μM PG at 24 h, only 800 and 1600 μM PG significantly elevated the percentage of GSH-depleted cells compared with control cells (Fig. 3A,B). Treatment with 1600 μM PG had the strongest effect, depleting intracellular GSH by 60% (Fig. 3A,B). Since CMFDA preferentially reacts with the reduced form over the oxidized form of GSH, this signal indirectly indicates a relatively higher reduced/oxidized ratio of GSH levels. Treatment with 200–1600 μM PG decreased intracellular GSH levels in HPF cells at 24 h, with the most significant decreases induced by 200 and 1600 μM PG (Fig. 3A,C). At the early time points of PG incubation (30–180 min), GSH levels decreased in HPF cells treated with all doses of PG at 30 min, and the gradual decrease lasted until 180 min (Fig. 3D). Compared to other doses, 1600 μM PG has stronger effects in decreasing GSH levels at 30, 45, 60, and 180 min (Fig. 3D).

### Effects of PG on SOD and catalase activity in HPF cells

**Figure 4:**
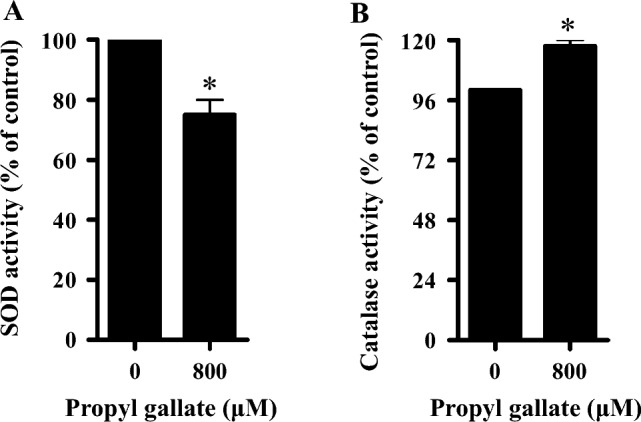
Effects of PG on the activity of SOD and catalase in HPF cells. Exponentially growing cells were treated with 800 μM PG for 24 h. (**A**,**B**) The graphs show the activity of SOD (**A**) and catalase in HPF cells (**B**). *P < 0.05 compared with the untreated control group.

Cellular redox status is regulated by many enzymes, including SOD and catalase. As shown in Fig. 4A, SOD activity was significantly downregulated in HPF cells treated with 800 μM PG at 24 h compared with that of control cells. By contrast, treatment with 800 μM PG significantly increased the activity of catalase in HPF cells at 24 h (Fig. 4B). At the concentration of 1600 μM PG, a significant cell death occurred, rendering it impractical to measure catalase and SOD activity.

### Effects of NAC and BSO on cell death and MMP (∆Ψm) in PG-treated HPF cells

**Figure 5:**
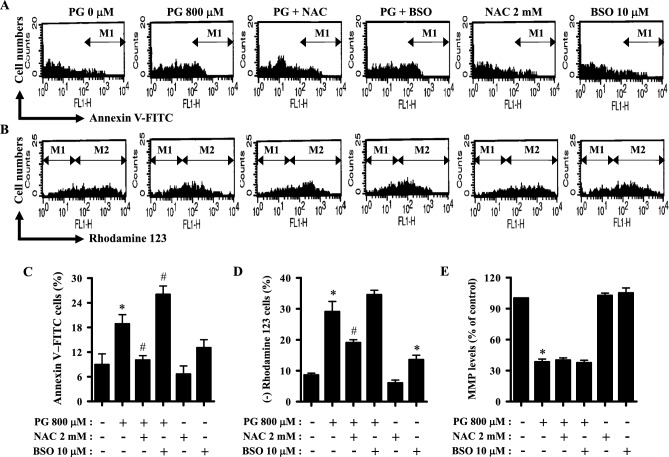
Effects of NAC and BSO on cell death and MMP (∆Ψ_m_) in PG-treated HPF cells. Cells undergoing exponential growth were pretreated with NAC (2 mM) or BSO (10 μM) for 1 h and then treated with 800 μM PG for 24 h. Annexin V-FITC and rhodamine 123 staining was performed in HPF cells and measured using a FAC Star flow cytometer. (**A**,**B**) Representative histograms of HPF cells stained with annexin V-FITC (**A**) or rhodamine 123 (**B**). M1 indicates annexin V-FITC-positive (**A**) and rhodamine 123-negative [MMP (ΔΨ_m_) loss] HPF cells (**B**). M2 indicates cells without MMP (ΔΨ_m_) loss. (**C**,**D**) Graphs show the percentage of annexin V-FITC-positive cells derived from M1 regions in A (**C**) and rhodamine 123-negative [MMP (ΔΨ_m_) loss] cells derived from M1 regions in B (**D**). (**E**) Graph displays the proportion of MMP (∆Ψ_m_) levels in HPF cells derived from M2 regions in (**B**), compared with control cells. *P < 0.05 compared with the untreated control group. ^#^*P* < 0.05 compared with cells treated with PG only.

Apoptosis and necrosis are cellular responses to cytotoxic agents, which are closely related to the loss of mitochondrial membrane potential (MMP; ∆Ψ_m_)^41,42^. Thus, cell death and MMP (∆Ψ_m_) in PG-treated HPF cells were evaluated using annexin V-FITC and rhodamine 123 fluorescence dyes. The effects of NAC and BSO on cell death and MMP (∆Ψ_m_) in PG-treated HPF cells were examined at 24 h. HPF cells were pretreated with NAC and BSO for 1 h before exposure to 800 μM PG, a concentration suitable for detecting changes in cell death and MMP (∆Ψ_m_) in the presence or absence of NAC or BSO. According to unpublished data, 100–200 μM PG did not significantly increase the number annexin V-positive HPF cells, but the number increased after incubation with 400–1600 μM PG. As shown in Fig. 5A,C, PG treatment increased the number of apoptotic HPF cells by 18%, a significant change. Although NAC significantly decreased the number of apoptotic cells in PG-treated HPF cells, BSO significantly increased this value in these cells (Fig. 5A,C). In addition, NAC slightly decreased the basal number of apoptotic cells in the control HPF cells, whereas BSO increased the basal number of those cells (Fig. 5A,C). Furthermore, approximately 28% of PG-treated HPF cells exhibited MMP (ΔΨ_m_) loss (Fig. 5B,D). Although NAC significantly reduced MMP (ΔΨ_m_) loss in PG-treated HPF cells, BSO slightly exacerbated this loss (Fig. 5B,D). NAC slightly decreased the basal number of control HPF cells with MMP (ΔΨ_m_) loss, whereas BSO significantly increased the basal number of those (Fig. 5B,D). The MMP (ΔΨ_m_) level in PG-treated HPF cells at 24 h, excluding cells whose MMP (ΔΨ_m_) was absent, was about 35% compared to that in the control cells (Fig. 5B,E). Neither NAC nor BSO altered the MMP (ΔΨ_m_) levels in PG-treated or untreated HPF cells (Fig. 5B,E).

### Effects of NAC and BSO on ROS and GSH levels in PG-treated HPF cells

**Figure 6:**
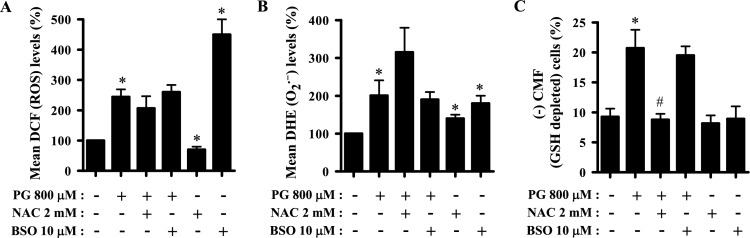
Effects of NAC and BSO on intracellular ROS and GSH levels in HPF cells. Cells undergoing exponential growth were pretreated with NAC (2 mM) or BSO (10 μM) for 1 h and then treated with 800 μM PG for 24 h. DCF (ROS), DHE (O_2_^**·**−^), and GSH levels were assessed using a FAC Star flow cytometer. (**A**,**B**) Graphs show the mean DCF (ROS) (**A**) and DHE (O_2_^**·**−^) levels in HPF cells (**B**), presented as a percentage of those in control cells. (**C**) Graph shows the percentage of CMF-negative (GSH-depleted) cells in HPF cells. *P < 0.05 compared with the untreated control group. ^#^*P* < 0.05 compared with cells treated with PG only.

The effects of NAC and BSO on ROS and GSH levels were evaluated in HPF cells treated with 800 μM PG at 24 h. Neither NAC nor BSO significantly changed the total ROS levels in PG-treated HPF cells, although NAC caused a slight decrease in ROS levels (Fig. 6A). NAC significantly decreased basal ROS levels in the control HPF cells, whereas BSO strongly increased the basal levels (Fig. 6A). Furthermore, NAC appeared to enhance the increase of O_2_^**·**−^ level induced by PG treatment in HPF cells, whereas BSO did not affect the O_2_^**·**−^ level (Fig. 6B). Both NAC and BSO increased basal O_2_^**·**−^ levels in the control HPF cells (Fig. 6B). Moreover, NAC significantly decreased the proportion of GSH-depleted cells in PG-treated HPF cells, whereas BSO showed no effect (Fig. 6C). Neither NAC nor BSO affected the proportion of GSH-depleted cells in the control HPF cells (Fig. 6C).

### Effects of antioxidant-related siRNAs on ROS and GSH levels and cell death in PG-treated HPF cells

**Figure 7:**
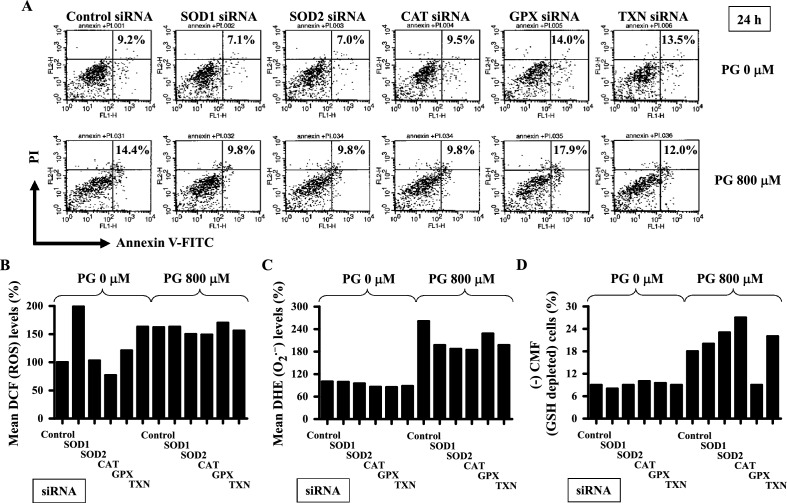
Effects of siRNAs against antioxidant genes on ROS and GSH levels and cell death in PG-treated HPF cells. HPF cells (at approximately 30–40% confluency) were transfected with either a nontargeting control siRNA or the indicated antioxidant-specific siRNAs. After 24 h, cells were treated with 800 μM PG for an additional 24 h. (**A**) Annexin V-FITC and PI staining in HPF cells was measured using a FAC Star flow cytometer. The numbers (%) in each figure indicate annexin V-FITC-positive cells, including both PI-negative and PI-positive cells. (**B**,**C**) Intracellular DCF (ROS) and DHE (O_2_^**·**−^) levels in HPF were measured using a FAC Star flow cytometer. Graphs show mean DCF (ROS) (**B**) and DHE (O_2_^**·**−^) levels (%) in HPF cells (**C**), presented as a percentage of those in control cells. (**D**) Intracellular GSH levels in HPF were measured using a FAC Star flow cytometer. Graph shows the percentage of CMF-negative (GSH-depleted) cells in HPF cells.

Furthermore, it was examined whether siRNAs against antioxidant genes (*SOD1, SOD2, CAT, GPX, or TXN*) affected ROS and GSH levels and cell death in HPF cells treated with 800 μM PG. The same siRNA sequences for *SOD1, SOD2, CAT, GPX, or TXN* were efficiently used in HPF cells^43,44^. As illustrated in Fig. 7A, the flow cytometry chart presented is a representative figure from the experiments. The proportions of apoptotic cells in PG-untreated and treated HPF cells were approximately 9% and 14% at 24 h, respectively. Administration of SOD1 or SOD2 siRNA slightly decreased the number of apoptotic cells in the control cells, whereas GPX or TXN siRNA appeared to increase the number (Fig. 7A). The administration of siRNA targeting SOD1, SOD2, or CAT resulted in the attenuation of cell death in PG-treated HPF cells, while GPX siRNA enhanced cell death (Fig. 7A). Additionally, PG treatment slightly led to a reduction in the proportion of apoptotic cells in HPF cells treated with TXN siRNA (Fig. 7A). siRNAs against SOD1, GPX, or TXN increased ROS levels in control HPF cells, while CAT siRNA decreased ROS levels at 24 h (Fig. 7B). siRNAs against SOD2, CAT, or TXN slightly downregulated the total ROS levels in PG-treated HPF cells (Fig. 7B). siRNAs against CAT, GPX, or TXN decreased O_2_^**·**−^ levels in control HPF cells (Fig. 7C). All siRNAs against antioxidants decreased O_2_^**·**−^ levels in PG-treated HPF cells (Fig. 7C). None of the tested siRNAs against antioxidants changed the proportion of GSH-depleted cells in control HPF cells (Fig. 7D). siRNAs against SOD2, CAT, or TXN appeared to augment the proportion of GSH-depleted cells in PG-treated HPF cells, whereas GPX siRNA decreased the proportion of those cells (Fig. 7D).

## Discussion

The in vivo or in vitro toxicological effects of PG have been previously evaluated^2,45–47^. However, there have been no reports on the cytotoxicological effects of PG on normal lung cells, such as fibroblast cells. Lung fibroblast cells are essential for maintaining the integrity of the alveoli and restoring injured tissues^34^. The present study focused on evaluating the effects of PG on ROS and GSH levels in normal HPF cells and further on examining the effects of NAC, BSO, and siRNAs against various antioxidants on ROS and GSH levels and cell death following PG treatment. PG induced cell death in HPF cells via caspase-independent apoptosis and/or necrosis (unpublished data). Moreover, treatment with PG (400–2000 μM) has been demonstrated to induce cell death in various cells by disrupting MMP (∆Ψ_m_)^6,11,26,48,49^. Consistent with this, treatment with 800 µM PG induced HPF cell death, accompanied by the loss of MMP (ΔΨ_m_). The proportion of HPF cells with MMP (∆Ψ_m_) loss after treatment with 800 μM PG was greater than that of dead cells. These results suggest that PG treatment primarily interrupts mitochondrial membranes, leading to subsequent stages of cell death.

PG can be an antioxidant^2,3,6,7,27^ or a pro-oxidant^6,10,30^. The present results showed that both ROS levels (as determined by DCF) and O_2_^**·**−^ levels (as determined by DHE) were increased in HPF cells treated with 100–800 μM PG at 24 h. Although PG significantly increased the activity of catalase in HPF cells, it did not reduce ROS levels. Interestingly, treatment with 1600 μM PG led to a high frequency of cell death in HPF cells (data not shown) at 24 h, without an increase in the levels of total ROS or O_2_^**·**−^. This discrepancy was likely due to the leakage of DCF and DHE dyes from the dead cells, leading to the failure to detect ROS. The ROS levels were increased in PG-treated HPF cells at the early time point of 30 min, with 800 and 1600 μM PG most dramatically increasing the ROS levels at 90 min. By contrast, all doses of PG decreased intracellular O_2_^**·**−^ levels at the early time point of 30 min, whereas only 1600 μM PG increased the O_2_^**·**−^ level at 45 min. All doses of PG increased O_2_^**·**−^ levels at 90 min. It is possible that PG initially increased ROS levels in HPF cells by affecting redox enzymes during early time points up to 60 min and subsequently elevated the levels of total ROS and O_2_^**·**−^ by damaging the mitochondria and altering the activity of redox enzymes at 90 min. Since mitochondrial O_2_^**·**−^ levels were increased in PG-treated HPF cells at 24 h, the increased O_2_^**·**−^ levels at 24 h likely resulted from the generation of O_2_^**·**−^ itself from the mitochondria. In addition, PG decreased the activity of SOD in HPF cells at 24 h. It is conceivable that the reduction of SOD activity results in a slower transformation from O_2_^**·**−^ to H_2_O_2_, consequently leading to the accumulation of O_2_^**·**−^ in cells. These results suggest that the tested doses of PG exhibited pro-oxidant effects, increasing intracellular ROS levels in HPF cells. Higher doses of PG at 24 h are likely to have both pro-oxidant and pro-apoptotic effects. Furthermore, PG caused the loss of MMP (ΔΨ_m_) in HPF cells. It was also reported that PG induces DNA strand breaks in A549 lung carcinoma cells^50^. Taken together, these data suggested that PG treatment induced HPF cell death via oxidative stress by increasing levels of total ROS, especially O_2_^**·**−^.

As a well-known antioxidant, NAC prevents cell death in PG-treated HeLa cervical cancer and endothelial cells, but increases ROS levels^6,27^. Similarly, NAC significantly prevented cell death and MMP (ΔΨ_m_) loss in PG-treated HPF cells, but did not significantly decrease ROS levels and even increased O_2_^**·**−^ levels. However, NAC alone significantly decreased basal ROS levels but increased the basal O_2_^**·**−^ levels in the control HPF cells. Moreover, NAC has been shown to decrease cell death and MMP (ΔΨ_m_) loss in arsenic trioxide- and MG132-treated HPF cells along with the downregulation of ROS levels^43,44^. BSO, a GSH synthesis inhibitor, showed a strong enhancement in cell death and MMP (ΔΨ_m_) loss in PG-treated HPF cells without increasing the levels of total ROS and O_2_^**·**−^. By contrast, BSO increased cell death and MMP (ΔΨ_m_) loss in HPF cells treated with arsenic trioxide and MG132 along with the upregulation of ROS levels^43,44^. BSO alone induced cell death and MMP (ΔΨ_m_) loss in control HPF cells and largely increased the levels of total ROS and O_2_^**·**−^. Thus, an increased ROS level induced by BSO treatment alone is tightly related to HPF cell death. PG is an antioxidant used to downregulate ROS levels in BSO-treated HPF cells. The diverse antioxidant or pro-oxidant effects observed with PG, NAC, BSO, and their combinations across various cell types, including both cancer and normal cells, may be attributed to the distinct baseline activities of mitochondria and antioxidant enzymes present in each specific cell type.

The current findings indicate that that 800 μM PG slightly increased the number of apoptotic cells, as well as the levels of total ROS and O_2_^**·**−^ in HPF cells treated with control siRNA. However, the percentage of apoptotic cells in PG-treated HPF cells was lower than anticipated when compared with that in HPF cells treated with control siRNA. This discrepancy might be attributed to the presence of LipofectAMINE 2000 agent in the medium and variations in cell seeding conditions, potentially influencing the effects of PG on cell death. Antioxidant proteins, especially SOD2 and TXN, have been known to stimulate cell proliferation and promote resistance to anti-growth agents^51–53^. Thus, downregulation of SOD2 or TXN may render cells sensitive to cytotoxic drugs. However, the present results showed that siRNAs against SOD1, SOD2, CAT, or TXN attenuated, instead of enhancing, PG-induced cell death in HPF. In addition, the effects of PG in inducing cell death were lower in HPF cells treated with each siRNA against antioxidants than those treated with control siRNA. Moreover, PG treatment decreased the percentage of apoptotic cells in HPF cells treated with TXN siRNA. The mechanism underlying the effects of SOD1, SOD2, CAT, and TXN on PG-induced HPF cell death remains to be further investigated. Changes in cell death in PG-treated HPF cells following siRNA silencing of antioxidant genes occurred without changes in total ROS levels, but was accompanied by the downregulation of the O_2_^**·**−^ levels. Therefore, the alteration of PG-induced HPF cell death by antioxidant-related siRNAs is not strongly associated with changes in ROS levels. Additionally, GPX or TXN siRNA increased apoptosis in untreated control HPF cells along with increases in total ROS levels, but not in O_2_^**·**−^ levels, whereas SOD1 or SOD2 siRNA decreased apoptosis in the control cells without decreasing the total ROS and O_2_^**·**−^ levels. These results suggest that the downregulation of each antioxidant protein by its corresponding siRNA influences ROS levels and cell death differently.

Intracellular GSH level is negatively correlated with progression to cell death^35,54,55^. Treatment with PG at concentrations ranging from 100 to 800 μM for 24 h induces depletion of GSH in HeLa cervical cancer cells^6^ and endothelial cells^27^. Moreover, PG at concentrations from 200 to 1600 μM for 24 h also depletes intracellular GSH in Calu-6 and A549 lung cancer cells^33^. Similarly, the current result showed that 800 or 1600 μM PG increased the number of GSH-depleted cells at 24 h. NAC effectively prevented GSH depletion in PG-treated HPF cells, consistent with its role as a GSH precursor. Surprisingly, BSO, a GSH synthesis inhibitor^56^, did not increase GSH-depleted cells. This unexpected result suggests complex regulatory mechanisms. BSO inhibits gamma-glutamylcysteine synthetase (γ-GCS), the first enzyme in GSH synthesis. Possible explanations include PG-induced activation of compensatory GSH synthesis pathways or alterations in GSH utilization, regeneration, or transport. This outcome underscores the intricate nature of cellular responses to oxidative stress, necessitating further investigation into the molecular mechanisms involved in GSH regulation during PG treatment and BSO inhibition. Treatment with 10 μM BSO alone increased cell death in control HPF cells without depleting GSH. In addition, GPX or TXN siRNA induced cell death in control HPF cells without increasing GSH depletion. Moreover, SOD1, SOD2, CAT, or TXN siRNA did not decrease GSH depletion in PG-treated HPF cells, but increased the number of GSH-depleted cells. However, GPX siRNA decreased the proportion of GSH-depleted cells among PG-treated HPF cells. Taken together, these data suggest that the intracellular GSH level plays a role in PG-induced cell death, although changes in its level alone are not sufficient for accurately predicting cell death. Notably, the GSH level in PG-treated HPF cells, except those CMF-negative cells, was generally decreased at early time points of 30–180 min and 24 h. The decreased GSH level probably resulted from its rapid consumption when reducing ROS levels at each corresponding time point.

In conclusion, PG induced cell death and MMP (ΔΨ_m_) loss in HPF cells, accompanied by increased ROS levels and GSH depletion. NAC treatment attenuated PG-induced cell death with decreased GSH depletion, while BSO increased cell death without altering ROS generation or GSH depletion. Additionally, siRNA silencing of SOD1, SOD2, or CAT attenuated cell death in PG-treated HPF cells, whereas silencing of GPX enhanced cell death, revealing association not tightly related to ROS or GSH levels. The findings from the current study offer valuable insights into the cytotoxic and molecular effects of PG on normal lung cells, especially HPF cells, by influencing ROS and GSH levels. These findings hold promising potential for clinical applications and cancer treatment strategies, presenting a proactive and impactful avenue for further exploration in medical interventions.

## Data Availability

Data collected during the present study are available from the corresponding author upon reasonable request.

## Abbreviations

HPF: Human pulmonary fibroblast
PG: Propyl gallate
ROS: Reactive oxygen species
MMP (ΔΨ_m_): Mitochondrial membrane potential
SOD: Superoxide dismutase
CAT: Catalase
GSH: Glutathione
NADPH: Nicotinamide adenine dinucleotide phosphate
GPX: GSH peroxidase
TXN: Thioredoxin
TXNR: TXN reductase
H_2_DCFDA: 2′,7′-Dichlorodihydrofluorescein diacetate
DHE: Dihydroethidium
CMFDA: 5-Chloromethylfluorescein diacetate
NAC: N-acetyl cysteine
BSO: L-buthionine sulfoximine
FBS: Fetal bovine serum
PI: Propidium iodide
FITC: Fluorescein isothiocyanate
siRNA: Small interfering RNA
